# A Novel Manno-Oligosaccharide Binding Protein Identified in Alkaliphilic *Bacillus* sp. N16-5 Is Involved in Mannan Utilization

**DOI:** 10.1371/journal.pone.0150059

**Published:** 2016-03-15

**Authors:** Yajian Song, Jinshan Li, Shan Meng, Liang Yin, Yanfen Xue, Yanhe Ma

**Affiliations:** 1 State Key Laboratory of Microbial Resources, Institute of Microbiology, Chinese Academy of Sciences, Beijing, 100101, China; 2 Key Laboratory of Industrial Fermentation Microbiology of the Ministry of Education, Tianjin Key Laboratory of Industrial Microbiology, College of Biotechnology, Tianjin University of Science and Technology, Tianjin, 300457, China; 3 Tianjin Institute of Industrial Biotechnology of Chinese Academy of Sciences, Tianjin, 300308, China; 4 National Engineering Laboratory for Industrial Enzymes, Tianjin Institute of Industrial Biotechnology, Chinese Academy of Sciences, Tianjin, 300308, China; Russian Academy of Sciences, Institute for Biological Instrumentation, RUSSIAN FEDERATION

## Abstract

ManH, a novel substrate-binding protein of an ABC transporter, was identified from the mannan utilization gene cluster of *Bacillus* sp. N16-5. We cloned, overexpressed, and purified ManH and measured its binding affinity to different substrates by isothermal titration calorimetry. ManH binds to mannotriose, mannotetraose, mannopentose, and galactosyl-mannotriose with dissociation constants in the micromolar range. Deletion of *manH* led to decreased growth ability of the strain when cultivated in medium with manno-oligosaccharides or mannan as the carbon source. ManH belongs to a manno-oligosaccharide transporter and plays an important role in mannan utilization by *Bacillus* sp. N16-5.

## Introduction

With the consumption of fossil fuels, exploitation of alternative renewable energy resources becomes increasingly necessary [1,2]. Plant biomass, which is mainly composed of cellulose, hemicellulose, and lignin, is the most abundant natural resource available for the production of biofuels [3,4,5]. The natural degradation of plant biomass is carried out mainly by microorganisms [6,7]. In this process, cellulose and hemicellulose are initially degraded into mono-, di-, and oligosaccharides, and sequentially transported into cells for further degradation and utilization [3,8]. Numerous extracellular and intracellular hydrolytic enzymes from microorganisms have been identified and extensively studied, but relatively little research has been conducted on the associated transport processes.

ABC (ATP binding cassette) transporter systems have been detected in all genera of the three kingdoms of life [9]. They are responsible for the uptake and efflux of solutes across the cell membrane, coupled to the energy release from ATP hydrolysis. Typically, ABC transporters consist of two transmembrane domains (TMDs), two nucleotide-binding domains (NBDs), and an extracellular substrate-binding domain (SBD), which binds and presents the substrate to the transporter channel [10]. ABC transporters that mediate the uptake of sugars are classified into carbohydrate uptake transporters 1 (CUT1) and carbohydrate uptake transporters 2 (CUT2). CUT1 family transporters have two different TMDs and two identical NBDs, which form a homodimer, while CUT2 family transporters have two identical TMDs, which form a homodimer and a fusion of two different NBDs. It has been reported that members of the CUT1 family transport a variety of di- and oligosaccharides, glycerol-phosphate, and polyols, while transporters belonging to the CUT2 family transport only monosaccharides [11].

Few ABC transporters participating in the uptake of hemicellulosic oligosaccharides have been reported in Gram-positive bacteria [8]. Among them, XynEF and BxlEFG, found respectively in *Geobacillus stearothermophilus* [12] and *Streptomyces thermoviolaceus* [13], were identified as transporters of xylo-oligosaccharides; AraNPQ and AraEFG, identified in *Bacillus subtilis* [14] and *G*. *stearothermophilus* [8], respectively, were responsible for the transport of arabino-oligosaccharides; CpMnBP1 identified in *Caldanaerobius polysaccharolyticus* was reported to be a SBD of a manno-oligosaccharide transporter and its co-crystal structures with bound substrate have been determined [15].

Mannan is a polysaccharide comprising linear or branched polymers derived from monosaccharides such as D-mannose, D-galactose, and D-glucose [16], and it is one of the major components of hemicellulose in the cell wall of plants. *Bacillus* sp. N16-5 is a Gram-positive hemicellulolytic alkaliphile that exhibits significant growth ability on natural mannan substrates [17]. In a previous study, we found a 16.6-kb sequence encoding 12 genes was dramatically upregulated at the transcriptional level when *Bacillus* sp. N16-5 was cultivated with locust bean gum as the sole carbon source, which strongly suggested that this gene cluster participates in mannan utilization [18]. Sequence similarity analysis revealed that the cluster contains one SBD and two TMDs of a sugar ABC transporter, which led us to the assumption that those three open reading frames form a CUT1 family ABC transporter for manno-oligosaccharide transport. To better understand the function of this ABC transporter, the substrate of the SBD was investigated here, along with the transporter’s physiological role in mannan utilization by *Bacillus* sp. N16-5.

## Materials and Methods

### Plasmids, strains, chemicals, and growth conditions

Table 1 lists the plasmids and bacterial strains used in this study. *Escherichia coli* DH5α and BL21(DE3) were grown in LB medium at 37°C and 50 μg/mL kanamycin was added to select plasmid transformants. *Bacillus* sp. N16-5 was cultivated in Horikoshi-II medium [19] in aerobic conditions at 37°C in shaken flasks. SA5 medium [20] was used in protoplast regeneration of *Bacillus* sp. N16-5 and neutral complex medium (NCM) [21] was used in deletion mutant construction.

**Table 1:** Bacterial strains and plasmids.

Strain or plasmid	Genotype or description	Source or reference
**Strain**		
*E*. *coli* DH5α	F^-^, φ 80d*lacZ* ΔM15, Δ(*lacZYA* -*argF*) U169, *deoR*, *recA1*, *endA1*, *hsdR17* (*rK*^-^, *mK*^*+*^), *phoA*, *supE44*, *λ*^*-*^, *thi -1*, *gyrA96*, *relA1*	Invitrogen
*E*. *coli* BL21(DE3)	F^-^, *ompT*, *hsdS*_*B*_(r_B_^-^ m_B_^-^), *gal*, *dcm* (DE3)	Novagen
*Bacillus* sp. N16-5 (CGMCC No.0369)	A facultative alkaliphilic strain producing multiple extracellular hydrolases	Lab storage
**Plasmid**		
pMD18-T	Cloning vector, Amp^r^	TaKaRa
pET-28a(+)	Expression vector, Kan^r^	Novagen
pNNB194	Shuttle vector between *E*. *coli* and *Bacillus* sp., Amp^r^, Erm^r^	[15]
pMK4	Shuttle vector between *E*. *coli* and *Bacillus* sp., Amp^r^, Cm^r^	[22]
pMK4A	pMK4 derivative carrying 500 bp *manA* upstream region, Amp^r^, Cm^r^	This work
pMK4AH	pMK4 derivative carrying 500 bp *manA* upstream region and *manH*, Amp^r^, Cm^r^	This work

All substrates (mannobiose, mannotriose, mannotetraose, mannopentose, galactosyl-mannotriose, xylotriose, and xylotetraose) were purchased from Megazyme (Wicklow, Ireland). Locust bean gum was purchased from Sigma Chemical Co. (St. Louis, Mo, USA). All other chemicals were commercially available and of analytical grade.

### Cloning, expression, and purification of ManH

The gene encoding ManH, which lacks an N-terminal signal peptide sequence, was amplified by PCR using genomic DNA of *Bacillus* sp. N16-5 as the template with sense primer manH-f containing a *Nco*I site, and antisense primer manH-r containing a *Bam*HI site. All the oligonucleotides used for PCR amplifications are listed in Table 2. The purified PCR product was digested with *Nco*I and *Bam*HI and ligated into pET-28a. Sequentially, the resultant recombinant plasmid was transformed into *E*. *coli* DH5α for cloning and then isolated and transformed into *E*. *coli* BL21(DE3) for protein expression.

**Table 2:** Oligonucleotides used in this study.

Name	Sequence	Description
manH-f	5’-ATGCCCATGGATGAGAATACTGGCGGTAATA-3’	*manH* coding sequence, cloning and expression
manH-r	5’-CGTAGGATCCTTATTCTATACCTTCAAGGCTTC-3’	*manH* coding sequence, cloning and expression
manH_D1	5’-GTTGTGGTGATGGCGTTT-3’	*manH* upstream region, deletion of *manH*
manH_D2	5’-AGAAAATGAACAAAAAATTGTGGGTGTCGATTTAAACTTAAACAATAA-3’	*manH* upstream region, deletion of *manH*
manH_D3	5’-TTATTGTTTAAGTTTAAATCGACACCCACAATTTTTTGTTCATTTTCT-3’	*manH* downstream region, deletion of *manH*
manH_D4	5’-ACAAATGTTCAAGAGCAAAC-3’	*manH* downstream region, deletion of *manH*
manA_Cf	5’-ATGCGTCGACGGACTTGCGGATCGAC-3’	*manA* upstream region, *manA* upstream region and *manH* fusions, complementation
manA_Cr	5’-CAACCACAATTTTTTGTTCATGTTATTACTCCTCCTTGTGGG-3’	*manA* upstream region, *manA* upstream region and *manH* fusions, complementation
manH_Cf	5’-CCCACAAGGAGGAGTAATAACATGAACAAAAAATTGTGGTTG-3’	*manH* coding sequence, *manA* upstream region and *manH* fusions, complementation
manH_Cr	5’-ATGCCCCGGGTTATTCTATACCTTCAAGGCTTC-3’	*manH* coding sequence, *manA* upstream region and *manH* fusions, complementation
manA_Cr2	5’-ATGCCCCGGGGTTATTACTCCTCCTTGTGGG-3’	*manA* upstream region (work with manA_Cf), complementation negative control

*E*. *coli* BL21(DE3) harboring the recombinant plasmid was initially cultivated aerobically at 37°C in LB medium containing 50 μg/mL kanamycin. When the cell density reached an OD_600_ of 1.0, 1 mM isopropyl-*β*-D-1-thiogalactopyranoside was added into the broth for induction of expression and the culture temperature was changed to 25°C. After overnight induction, the cells were harvested by centrifugation (5,000 × *g*, 4°C, 10 min), and the pellet was resuspended in 20 mM Tris-HCl (pH 8.0) followed by disruption by ultrasonic treatment. After centrifugation at 12,000 × *g* for 15 min, the supernatant of the cell lysate was filtered through a 0.22-μm filter and applied to a 6-mL RESOURCE™ Q column (GE Healthcare, USA) mounted on an AKTA-explorer FPLC (GE Healthcare, USA) for purification. The purification was carried out according to the instructions for use of the RESOURCE™ Q column at pH 8.0 and a flow rate of 2 mL/min. 20 mM Tris-HCl was used as the starting buffer and 20 mM Tris-HCl with 1 M NaCl was used as the elution buffer. The desired fractions were collected when the ionic strength was between 0.45 and 0.55 M NaCl.

Protein was visualized by sodium dodecyl sulfate polyacrylamide gel electrophoresis, and the concentration was determined using a Bio-Rad protein assay kit (Bio-Rad, USA).

### Microcalorimetry titration analysis

The ability of ManH to bind different sugars was determined using an isothermal titration calorimeter (NANO-ITC 2G; TA Instruments, USA) at 30°C. The buffer for purified protein was replaced by 20 mM Tris-HCl (pH 8.0) containing 200 mM NaCl using a desalting column (GE Healthcare). Protein samples for isothermal titration calorimetry (ITC) analysis were prepared in the concentration range of 20–96 μM. Ligand solutions (mannobiose, mannotriose, mannotetraose, mannopentose, galactosyl-mannotriose, xylotriose, and xylotetraose, mannose, glucose, fructose, and galactose) were prepared in the same buffer at concentrations from 10- to 15-fold higher than that of the target protein. When the affinity between galactosyl-mannotriose and protein was tested, aliquots (10 μL) of the ligand solution were added by means of a 250-μL rotating stirrer-syringe to the reaction cell containing 1.41 mL protein solution. For the other sugars, aliquots (5 μL) of ligand solution were added via a 100-μL rotating stirrer-syringe. Binding parameters such as the binding constant (*K*_a_ [/M]) and the binding enthalpy (Δ*H*_a_ [kcal/mol]) were evaluated by fitting the experimental binding isotherms using NanoAnalyze™ Software (TA Instruments). The “independent” model was chosen for the data fitting; in this model, the protein has one or more binding sites, and each site is thermodynamically identical. The logic of the model including functions and constants was described in the software.

### Plasmid and mutant construction

The *E*. *coli/B*. *subtilis* shuttle vector pNNB194 with a temperature-sensitive *B*. *subtilis* origin of replication [23] was used as the backbone for gene deletion. Mutant strain Δ*manH* was obtained by removing the entire coding sequence of ManH without introduction of an antibiotic-resistant gene. In the first step, the 800-bp DNA fragments located directly upstream and downstream of *manH* were generated and fused by PCR. The fusion product was cloned into vector pMD18-T and subsequently inserted into pNNB194 between *Bam*HI and *Sal*I restriction sites. The resulting plasmid was introduced into *Bacillus* sp. N16-5 protoplast as described previously [20], and the transformants were selected on SA5 plates containing 0.5 μg/mL erythromycin at the permissive temperature of 34°C. A single transformant was then inoculated into NCM medium supplemented with erythromycin and cultivated in aerobic conditions at 37°C in a tube. The culture was then spread on a NCM plate containing erythromycin and grown at 45°C to select for plasmid integration into the chromosome. To promote homologous recombination and plasmid loss from the locus targeted for deletion, colonies from the NCM plate were inoculated into Horikoshi-II medium and grown at 37°C without selective pressure. The cultures were then spread onto Horikoshi-II plates, and single colonies were patched to NCM plates with or without erythromycin. Colonies that were erythromycin sensitive were screened by PCR to identify the *manH* deletion mutant Δ*manH*.

*E*. *coli/B*. *subtilis* shuttle vector pMK4 was used for gene complementation experiments. The 500-bp upstream region of *manA* and the entire sequence of *manH* was amplified and fused by PCR. The fusion product was then inserted into pMK4 between *Sac*I and *Xma*I restriction sites to generate plasmid pMK4AH. pMK4AH was transformed into the Δ*manH* protoplast and the transformants were selected on SA5 plates containing 2.5 μg/mL chloramphenicol at 37°C to obtain the ManH complementation strain, Δ*manH*-pMK4AH. The 500-bp *manA* upstream region was also amplified and separately inserted into pMK4 to generate plasmid pMK4A. pMK4A was introduced into Δ*manH* to obtain the complementation negative control strain, Δ*manH*-pMK4A. Strains Δ*manH*-pMK4 and WT-pMK4 were generated by introducing the parental pMK4 into Δ*manH* and wild-type *Bacillus* sp. N16-5, respectively.

### Cell density determination

To determine the growth of strains WT-pMK4, Δ*manH*-pMK4, Δ*manH*-pMK4AH, and Δ*manH*-pMK4A with mannose and mannan as carbon source, cells were cultivated at 37°C in shaken flasks containing 50 mL medium (5 g peptone, 1 g K_2_HPO_4_·3H_2_O, 0.2 g Mg_2_SO_4_·7H_2_O, 0.1 g yeast extract, and 5 g locust bean gum per liter). 5 mL Na_2_CO_3_ solution (10%) was added to 50 mL medium after separate sterilization and 2.5 μg/mL chloramphenicol was also added separately. To determine the growth of strains WT-pMK4, Δ*manH*-pMK4, Δ*manH*-pMK4AH, and Δ*manH*-pMK4A with mannobiose and manno-oligosaccharide as the carbon source, the strains were cultivated at 37°C in 5-mL tubes containing 1.5 mL medium, which included 5 g mannobiose or manno-oligosaccharide per liter instead of locust bean gum. Growth curves were determined by measuring cell density of the cultures at 600 nm (OD_600_) every 3 h. Optical density was measured using a 96-well plate spectrometer (SpectraMax 190; Molecular Devices, USA) and 200 μL liquid was added to each well of the microplate.

### Bioinformatic analysis

Similar sequences of ManH were searched in the non-redundant protein sequence database using blastp (http://blast.ncbi.nlm.nih.gov/Blast.cgi) with default parameters. The theoretical molecular weight and theoretical isoelectric point (*pI*) were predicted using Compute pI/Mw (http://web.expasy.org/compute_pi/).

### Nucleotide sequence

The 16,598-bp sequence of the mannan utilization region from *Bacillus* sp. N16-5 has been deposited to GenBank with the accession number KU644713. The protein_id of ManH is AML27057.

## Results and Discussion

### Sequence analysis of the gene cluster for mannan utilization from *Bacillus* sp. N16-5

**Fig 1:**
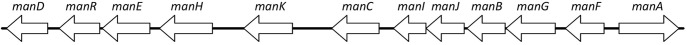
Gene cluster for mannan use in *Bacillus* sp. N16-5.

Fig 1 shows the structure of the 16.6-kb mannan utilization gene cluster; the predicted function of each open reading frame is listed in Table 3. This gene cluster includes 12 genes which were designated *manR*, *H*, *I*, *J*, *A*, *B*, *C*, *D*, *E*, *F*, *G*, and *K* respectively. According to sequence alignments, ManH was annotated as the SBD of a sugar ABC transporter, and ManI and ManJ were annotated as TMDs of a sugar ABC transporter. No homolog of ManH, I, or J has been characterized. Moreover, ManH has no significant sequence similarity with CpMnBP1, the only reported SBD of a manno-oligosaccharide transporter, which suggests that ManH is likely to exhibit different features from CpMnBP1. As ManI and ManJ encode two different TMDs, we suggest that the three proteins comprise a CUT1 family ABC transporter. No NBD encoding gene was found in this gene cluster. However, it has been reported that the NBD can be shared among different ABC transporters, and the absence of its encoding gene from a gene cluster is a common occurrence [24,25,26].

**Table 3:** Predicted function of genes from the mannan utilization gene cluster of *Bacillus* sp. N16-5.

Category and gene	Predicted function
**Transcriptional regulator**	
*manR*	LacI family transcriptional regulator
**Enzymes**	
*manA*	Mannan endo- β -1,4-mannanase
*manB*	Endo- β -1,4-mannanase
*manC*	α-Galactosidase
*manD*	Glycosidase
*manE*	Glycosidase
*manF*	Acetylxylan esterase
*manG*	N-Acylglucosamine 2-epimerase
**Sugar transporter**	
*manH*	ABC transporter: solute binding protein
*manI*	ABC transporter: transmembrane domains
*manJ*	ABC transporter: transmembrane domains
**Others**	
*manK*	Hypothetical protein

### Substrate specificity of ManH

**Fig 2:**
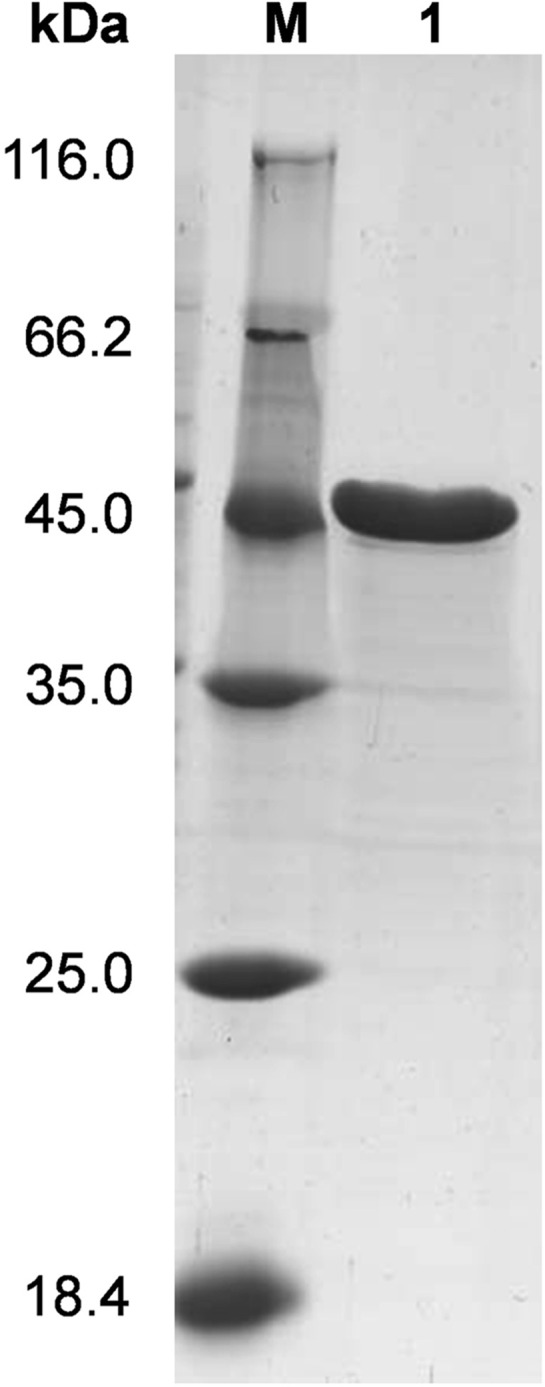
SDS-PAGE analysis of purified ManH. Lane M: protein markers (values in kDa on the left). Lane 1: purified ManH protein.

**Fig 3:**
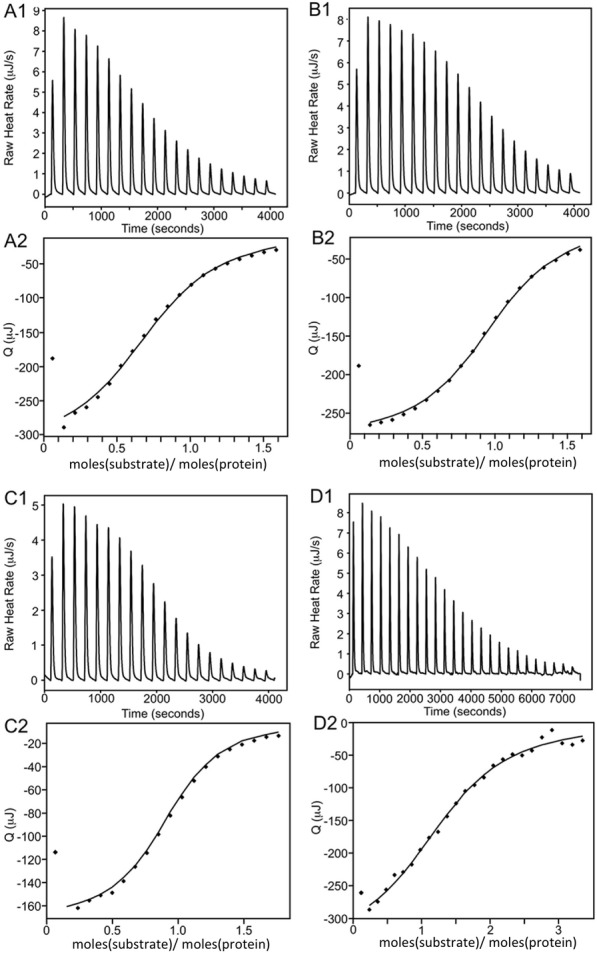
Isothermal calorimetric titration curves of the interactions of ManH with mannotriose, mannotetraose, mannopentose, and galactosyl-mannotriose. (A1) Calorimetric titrations of 0.03 mM ManH with 0.45 mM mannotriose. (A2) Integrated injection heats from A1. (B1) Calorimetric titrations of 0.03 mM ManH with 0.45 mM mannotetraose. (B2) Integrated injection heats from B1. (C1) Calorimetric titrations of 0.018 mM ManH with 0.3 mM mannopentose. (C2) Integrated injection heats from C1. (D1) Calorimetric titrations of 0.086 mM ManH with 1 mM galactosyl-mannotriose. (D2) Integrated injection heats from D1.

ManH without an N-terminal signal peptide sequence was cloned and expressed in *E*. *coli* BL21(DE3). As the theoretical *pI* of ManH was predicted to be 3.85, strong anion exchange chromatography was used for purification. As shown in Fig 2, ManH with theoretical molecular weight of 42kDa was highly purified after one-step anion exchange. ITC, which directly measures the thermodynamic parameters of the interaction between two molecules in solution [27], was used to evaluate the binding affinity of ManH to different monosaccharides and oligosaccharides. Among the tested sugars, mannotriose, mannotetraose, mannopentose, and galactosyl-mannotriose, exhibited typical binding curves with ManH. The titration curves and the thermodynamic parameters are summarized in Fig 3 and Table 4. The association constants (*K*_a_) of ManH and mannotriose, mannotetraose, mannopentose, and galactosyl-mannotriose were determined as 26.5×10^4^, 45.3×10^4^, 122.0×10^4^, and 5.2×10^4^ M^-1^ respectively. No obvious binding curves were observed for the other tested sugars, which suggested that there were no specific interactions between ManH and these substrates in our experimental conditions. It was reported that CpMnBP1 from *C*. *polysaccharolyticus* could bind tightly to mannobiose [15], but ManH had no binding affinity with mannobiose. The ITC data indicated that the substrate of ManH is manno-oligosaccharide, and we concluded that the transporter to which ManH belongs is involved in manno-oligosaccharide uptake.

**Table 4:** Binding of ManH to sugars: thermodynamic parameters and dissociation constants.

Sugar	*K*_a_ (×10^4^ M^-1^)	*K*_d_ (1/*K*_a_, μM)	Δ*H*_a_ (kJ/mol)	TΔ*S*_a_ (kJ/mol)	Δ*G*_a_ (kJ/mol)
mannotriose	26.5±5.0	3.8	-136.7±7.1	-105.3	-31.5
mannotetraose	45.3±2.6	2.2	-109.4±21.3	-76.6	-32.8
mannopentose	122.0±16.3	0.8	-115.2±5.3	-79.9	-35.3
galactosyl-mannotriose	5.15±1.46	19.4	-33.5±2.1	-6.1	-27.3

### Growth behavior of *manH* deletion mutant on mannose, mannobiose, and manno-oligosaccharides

The ITC data revealed that ManH forms part of a manno-oligosaccharide ABC transporter, so its absence should affect the utilization of manno-oligosaccharides by cells but not affect mannose and mannobiose usage. To confirm this, and to better understand the physiological role of ManH, we investigated the growth behavior of a *manH* deletion mutant in medium containing mannose, mannobiose, mannotriose, mannotetraose, and mannopentose, respectively.

A *manH* deletion mutant strain, Δ*manH*, was constructed (see Materials and Methods). Upstream sequence of *manA* (500 bp) supposed to contain RBS (ribosome binding site) sequence and a mannan inducing promoter was fused with the *manH* gene and inserted into pMK4 to form pMK4AH; this plasmid was introduced into Δ*manH* to produce the complementation strain Δ*manH*-pMK4AH. Plasmid pMK4 carrying only the 500-bp *manA* upstream sequence was also introduced into Δ*manH* to construct the negative control complementation strain Δ*manH*-pMK4A. Meanwhile, parental pMK4 was introduced into the wild-type strain and Δ*manH* to generate WT-pMK4 and Δ*manH*-pMK4 to enable comparison of all strains in equivalent conditions.

**Fig 4:**
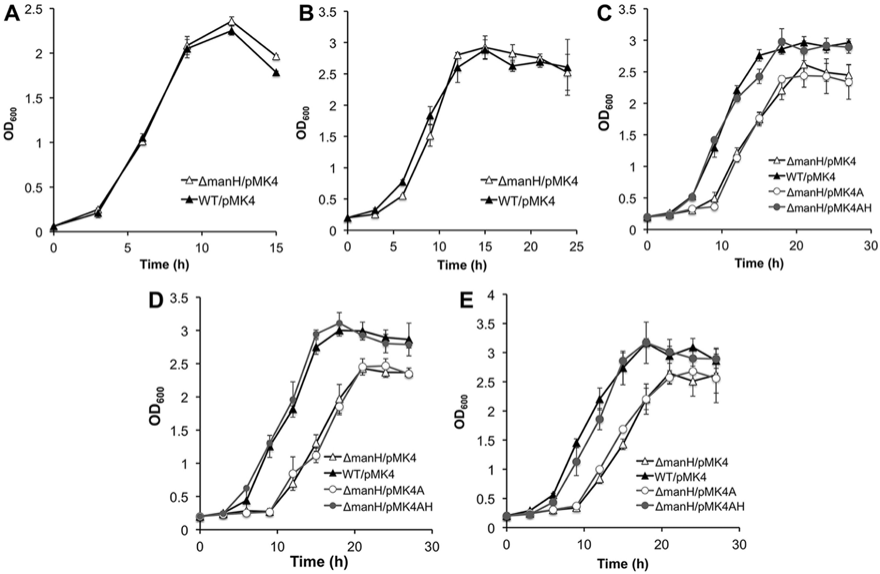
Growth curves of WT-pMK4, Δ*manH*-pMK4, Δ*manH*-pMK4A, and Δ*manH*-pMK4AH with manno-oligosaccharides as the substrate. (A) mannose; (B) mannobiose; (C) mannotriose; (D) mannotetraose; (E) mannopentose.

Cell growth behavior on different substrates was first compared between the *manH* deletion mutant and the wild-type. As Fig 4A and 4B show, Δ*manH*-pMK4 and WT-pMK4 had identical growth curves in medium containing mannose or mannobiose, demonstrating that the deletion of *manH* did not affect the utilization of these two substrates. However, the growth of Δ*manH*-pMK4 was less than that of WT-pMK4 when the strains were cultivated in medium with mannotriose, mannotetraose, or mannopentose as the carbohydrate resource. Δ*manH*-pMK4AH had restored growth ability, while Δ*manH*-pMK4A did not (Fig 4C, 4D and 4E). These data indicate that the deletion of *manH* decreased the manno-oligosaccharide utilization ability of *Bacillus* sp. N16-5, i.e., ManH participates in the use of manno-oligosaccharides.

It should be noted that although the growth of Δ*manH* was delayed when the carbon source was mannotriose, mannotetraose, or mannopentose, a relatively high amount of biomass was still obtained (around 80% of that observed for the wild-type strain). The extracellular mannanase ManA not only degrades mannan to manno-oligosaccharides but also to mannose and mannobiose [17], so we suggest that *Bacillus* sp. N16-5 (including strain Δ*manH*) can utilize the generated mannose and mannobiose via a specific transporter(s) to maintain growth. However, the growth of strain Δ*manH* was limited because of the absence of an oligosaccharide transporter.

### Manno-oligosaccharide transport participates in mannan utilization

**Fig 5:**
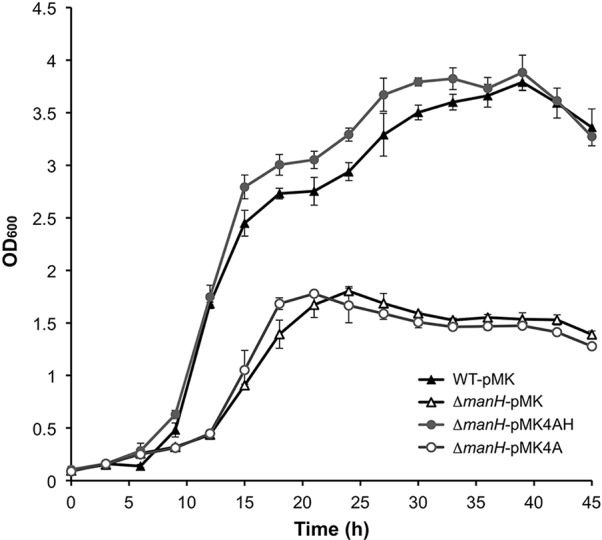
Growth curves of WT-pMK4, Δ*manH*-pMK4, Δ*manH*-pMK4A, and Δ*manH*-pMK4AH with locus bean gum as the substrate.

We cultivated strains Δ*manH*-pMK4, WT-pMK4, Δ*manH*-pMK4AH, and Δ*manH*-pMK4A in medium with locust bean gum as the carbon source. As Fig 5 shows, Δ*manH*-pMK4 exhibited remarkably decreased growth, manifested as a prolonged lag phase and only half-maximum cell density, compared with strain WT-pMK4. Strain Δ*manH*-pMK4AH exhibited similar growth behavior to WT-pMK4, while strain Δ*manH*-pMK4A did not restore the growth ability of Δ*manH*, and it showed a growth profile comparable to that of Δ*manH*-pMK4. These results demonstrate that deletion of *manH* greatly inhibited mannan utilization by *Bacillus* sp. N16-5, thus manno-oligosaccharide transport plays an important role in rapid and efficient mannan utilization.

In our previous study, the transcription of the mannan utilization gene cluster was found to be significantly induced by mannan and signal peptide prediction suggested that all the enzymes in the cluster were intracellular proteins except for ManA [18]. Based on this, a mannan utilization hypothesis was proposed: mannan is first randomly depolymerized by extracellular endo-β-1,4-mannanase (ManA); the generated manno-oligosaccharides are then taken up via an ABC transporter system (ManHIJ) and subsequently metabolized into monosaccharides by intracellular enzymes [18]. In this case, manno-oligosaccharides are degraded in the cytoplasm instead of extracellularly, which will reduce the necessary quantity of enzymes and increase the efficiency of hydrolysis. Similar hypothesis concerning polysaccharide use based on oligosaccharide transport has also been proposed in some other bacteria [8,15,25], but this is the first study proving that manno-oligosaccharide transport contributes to efficient mannan utilization.

## Conclusions

In this study, we report a novel manno-oligosaccharide-binding protein ManH, which is the SBD of an ABC transporter. The thermodynamic parameters of ManH binding to several substrates were determined using ITC. Gene deletion experiments proved that ManH affects manno-oligosaccharide utilization by *Bacillus* sp. N16-5. Moreover, the deletion of *manH* led to a dramatic decrease in cell growth ability when locust bean gum was the sole carbon source, which suggests an important role for oligosaccharide transport in efficient mannan utilization. The characterization of ManH will contribute to the identification of other proteins of this type and deepen our understanding of mannan utilization mechanisms in bacteria.
